# Appropriateness of malaria diagnosis and treatment for fever episodes according to patient history and anti-malarial blood measurement: a cross-sectional survey from Tanzania

**DOI:** 10.1186/s12936-018-2357-7

**Published:** 2018-05-21

**Authors:** Joanna Gallay, Dominic Mosha, Erick Lutahakana, Festo Mazuguni, Martin Zuakulu, Laurent Arthur Decosterd, Blaise Genton, Emilie Pothin

**Affiliations:** 1 Department of Epidemiology and Public Health, Swiss Tropical and Public Health Institute, P.O. Box 4002, Basel, Switzerland; 2 University of Basel, Basel, Switzerland; 3 Service and Laboratory of Clinical Pharmacology, University Hospital, Lausanne, Switzerland; 4 Ifakara Health Institute, Dar es Salaam, Tanzania; 5 Division of Infectious Diseases and Department of Community Health, University Hospital, Lausanne, Switzerland

**Keywords:** Malaria, Fever case management, Malaria diagnosis, RDT, Malaria treatment, Lumefantrine, Tanzania, Antimalarial drugs blood measurements

## Abstract

**Background:**

Monitoring the impact of case management strategies at large scale is essential to evaluate the public health benefit they confer. The use of methodologies relying on objective and standardized endpoints, such as drug levels in the blood, should be encouraged. Population drug use, diagnosis and treatment appropriateness in case of fever according to patient history and anti-malarials blood concentration was evaluated.

**Methods:**

A cross-sectional survey took place between May and August 2015 in three regions of Tanzania with different levels of malaria endemicity. Interviews were conducted and blood samples were collected by dried blood spots through household surveys for further anti-malarial measurements. Appropriate testing when individuals attended care was defined as a patient with history of fever being tested for malaria and appropriate treatment as (i) having anti-malarial in the blood if the test result was positive (ii) having anti-malarial in the blood if the person was not tested, and (iii) no anti-malarial in the blood when the test result was negative.

**Results:**

Amongst 6391 participants included in the anti-malarial analysis, 20.8% (1330/6391) had anti-malarial drug detected in the blood. Only 28.0% (372/1330) of the individuals with anti-malarials in their blood reported the use of anti-malarials within the previous month. Amongst all participants, 16.0% (1021/6391) reported having had a fever in the previous 2 weeks and 37.5% of them (383/1021) had detectable levels of anti-malarials in the blood. Of the individuals who sought care in health facilities, 69.4% (172/248) were tested and 52.0% (129/248) appropriately treated. When other providers were sought, 6% (23/382) of the persons were appropriately tested and 44.2% (169/382) appropriately treated. Overall, the proportion of individuals treated was larger than that being tested [47.3% (298/630) treated, 31.0% (195/630) tested].

**Conclusion:**

This study showed high prevalence of circulating anti-malarial drug in the sampled population. Efforts should be made to increase rapid diagnostic tests use at all levels of health care and improve compliance to test result in order to target febrile patients that are sick with malaria and reduce drug pressure. Objective drug measurements collected at community level represent a reliable tool to evaluate overall impact of case management strategies on population drug pressure.

## Background

The implementation of artemisinin-based combination therapy (ACT) and malaria rapid diagnostic tests (RDTs) has been a cornerstone in the management of fever cases. These tools are essential components of the current global malaria control strategy [1–3]. In Tanzania, a good coverage of preventive and curative interventions, including the distribution of insecticide-treated nets and the adoption of ACT as first-line therapy, has led to a decrease in the number of malaria cases of more than 75% between 2000 and 2015 [4]. Considerable efforts have been made to expand access to effective anti-malarials in the public and private sector. In 2014, Tanzania has reported that sufficient ACT medicines had been distributed across the country to treat all patients attending public health facilities [5]. Malaria RDTs have also been deployed to reach half of the population so far, and health workers have been trained in using them [4, 6]. Recent surveys have reported that availability of malaria testing was 83% in the public sector where more than 70% of the suspected cases were tested [4, 7]. This was not the case in the private sector where there was only 16% of testing available [7]. Diagnosis availability and compliance to diagnosis results are major factors to reach rational use of treatments. Although the Tanzania National Malaria Control Programme (NMCP) case management policies, recommended by the World Health Organization (WHO) requires parasitological confirmation of malaria prior to treatment for patients of all ages, there are concerns that many patients with malaria do not receive ACT while others suffering from non-malarial fever do. This indicates that treatments are not always targeted to those in need [2, 3, 6]. While under-treatment needs to be addressed with improved access to drugs, over-treatment due to non-availability of malaria tests or lack of expertise of clinicians in the management of non-malaria fevers has becomes a concern increasingly important, especially with global decrease in proportion of febrile illnesses due to malaria [8, 9]. Incorrect malaria prescriptions result in wastage of medication, delays in obtaining effective treatment for the true cause of illness, important drug pressure in the population [10] and hence emergence of parasite resistance to drugs [11].

Monitoring case management strategies and evaluating their impact are important activities to ensure that they confer the foreseen individual and public health benefit they are supposed to. To that end, interviews targeting caretakers of small children in community based cross-sectional surveys as well as data collection in health facilities or in the retail sector have been used to estimate levels of access to good quality drugs and the impact of RDTs use on drug prescription [5, 6, 12–14]. These studies inherently suffer from potential biases, such as recall bias and inaccurate reporting due to fear to be judged or fear of not being appropriately cared for [15]. They are also more likely to detect a positive effect since they are usually conducted in places where intense training and supervision have been undertaken, or are biased due to the Hawthorne effect [16]. Two studies conducted in Tanzania and in Cambodia showed that self-reported history is not reliable in terms of actual drug use. Indeed, 75% of patients presenting in a health facility in Tanzania and 50% in Cambodia had detectable concentrations of anti-malarials in the blood, although all stated that they did not take any drug in the previous month [17, 18]. Besides recourse to public health facilities, febrile patients often seek care in the private sector, and especially so among drug retailers that are usually prohibited to sell and perform RDT testing which is the case in Tanzania, except for registered accredited drug dispensing outlets (ADDOs) [5, 9, 10, 19, 20]. As a result, a considerable amount of patients are prescribed anti-malarial treatments presumptively. For all these reasons, the overall impact of the implementation of RDTs and ACT is difficult to assess precisely. There is a need to apply more rigorous and reliable methodologies to evaluate the appropriateness of case management for fever episodes at large scale. The aim of this study was to use anti-malarials blood levels as an objective and standardized endpoint to evaluate population drug use, to compare these results with self-reported history, and to assess diagnosis and treatment appropriateness in case of fever episode at population level.

## Methods

### Study design

This cross-sectional survey included three types of surveys conducted concurrently in randomly selected wards (which is the smallest administrative area and includes five to seven villages): (1) household-based surveys, (2) drug outlet-based surveys and (3) exit interviews in health facility-based surveys.

### Study areas and population

The surveys took place in 2015, after the rainy season in three regions of Tanzania: Mtwara, Mwanza and Mbeya with populations of 1 270 854, 2 772 509 and 2 707 410, respectively [21]. The IMPACT2 project [5], whose main objective was to assess the impact of the Affordable Medicines Facility-malaria (AMFm) initiative on the supply and demand of ACT medicines, served as a basis for the choice of the regions in this study. In this study performed in 2012, the level of malaria prevalence amongst all age groups was moderately high for Mtwara and Mwanza (17.4 and 16.1% respectively), and low for Mbeya (2.3%). Tanzania is an area of year-round malaria transmission, with a bimodal pattern, peaking after the rainy season. Each region includes urban and rural districts, although the populations are predominantly rural. Tanzania has four different administrative levels, the highest level being the region. Regions are divided into districts and these are sub-divided into divisions and further into wards. Fever case management in the public sector is provided by a network of regional and district hospitals as well as health centres and dispensaries at lower administrative levels [22]. The private sector includes for profit and not-for-profit facilities (hospital and clinics) and a drug outlets network which is mainly constituted of regulated and non-regulated drug shops, while registered pharmacies are almost exclusively located in major urban areas [23, 24].

### Study sampling

One urban and two rural districts were selected in each study region. Three wards were randomly selected proportionally to their population size in each district. In each urban ward, four streets and in each rural ward, two villages as well as two sub villages in each village were randomly selected. After obtaining the list of the households within each sub village/street, 20 households were randomly sampled for the household surveys. In each sampled household, up to six participants were randomly selected from the complete list of the household members until a total of 60 individuals per sub-village/street was reached, resulting in 240 individuals sampled per ward. All consenting individuals were eligible to participate. The exclusion criteria were individuals with a severe illness requiring immediate referral and those under 3 months of age.

### Data collection procedures

#### Household surveys

Interviews were conducted with a questionnaire in Swahili, first with the head of household. The questions also included information on time to the closest health facility and closest pharmacy or drug retailers. Randomly selected members were then asked about demographic information, history of fever in the previous 2 weeks as well as history of anti-malarial use in the previous months. Members who reported fever in the previous 2 weeks were asked about treatment-seeking behaviour including place where they sought care, information on malaria diagnosis testing and drugs received and ingested. Data was collected using electronic tablets with the help of the Open Data Kit collection tool (ODK). Each visited household was mapped using Global Positioning System. In addition, blood spots were collected from finger prick onto filter paper to assess the presence of malaria antigens and for further drug concentrations measurements.

#### Outlet surveys

Drug-outlet surveys were conducted in all private and public outlets surrounding and serving the selected villages/streets. These included small district hospitals, public and private health centres, dispensaries, pharmacies, registered ADDOs and non-registered drug retailers, general stores and kiosks. Following verbal consent of the most senior staff present at the moment of the survey, details about anti-malarials stocks and diagnostic tools (RDTs and microscope) available at the time of the visit were recorded.

#### Exit interviews of patients in health facilities

In each ward, the main health facilities (which included district hospitals, health centres and/or dispensaries) serving the surveyed villages were selected for the exit interviews. After completion of their consultation with health facility staff and after visiting the health facility pharmacy for possible treatment procurement, patients were interviewed and information were collected on demographics, administration of malaria diagnostic testing (microscopy or RDTs), test result and drug obtention. In addition, RDT was performed on site by the field investigators.

### Laboratory procedures

Capillary blood samples were taken by fingerprick on all subjects interviewed during the household surveys. One drop of blood was immediately used for RDTs analysis (ParaHIT-*f* test, Span diagnostic Ltd, Surat, India, detecting HRP-2 antigens) and four drops were applied on filter paper cards (FTA DMPK-B cards, Whatman, GE Healthcare). These were able to dry at room temperature for at least two hours before being placed in a specific bag with desiccant and stored in a − 10 °C freezer at the end of the day and finally transferred to a − 80 °C freezer within 1 month. Concentrations of seven anti-malarials and two metabolites, namely amodiaquine, *N*-desethyl-amodiaquine, lumefantrine, desbutyl-lumefantrine, mefloquine, chloroquine, quinine, sulfadoxine and pyrimethamine, were determined in the dried blood spots (DBS) samples by liquid chromatography coupled to tandem mass spectrometry (LC–MS/MS) [25, 26]. The LC–MS/MS platform enables to detect residual blood levels if the drug was taken up to 4 weeks prior to the analysis (given the long half-lives of the measured anti-malarials). Due the very short half-life of artemisinin compounds, and because they are rapidly degraded due to haemolysis with the current collection procedure, their analysis were not performed in these DBS samples.

### Definitions

Fever was defined as any illness with fever reported in the 2 weeks prior to the survey. Malaria infection was defined as a positive RDT result on the day of the survey. Anti-malarials identified during the outlet surveys were classified according to their active ingredients and drug formulation. For data analysis, outlets were considered to have RDTs and anti-malarials in stock if the study team observed at least one non-expired test or at least one complete non-expired treatment of any anti-malarial for any age/weight group. Individuals were considered having anti-malarials in the blood if at least one of the nine anti-malarials/metabolites were measured in their corresponding DBS sample, at a concentration equal or higher than the lower limit of quantification. The latter is the minimal concentration that confidently provides a bias and coefficient of variation within ± 20% [27]. For this analysis, dispensaries, public, private or mission health centres and small district hospitals were classified as “health facilities”. Pharmacies, registered (ADDOs) and non-registered drug retailers, general stores and kiosks were grouped as “non-health facility anti-malarial providers”. Traditional practitioner, neighbours, friends or even home (if medication available) were classified as “other places”. Appropriate diagnosis was defined as a patient with history of fever being tested for malaria (by RDT or microscopy) and appropriate treatment as having anti-malarial in the blood or not in agreement with RDT result. Treatment was also considered as being appropriate if a febrile individual was not tested and had anti-malarials detected in the blood, as per WHO guidelines when diagnostic testing is not possible [3].

### Data management and statistical analysis

Data was stored on the ODK Aggregate data repository at the end of each survey day. During each interview, key data such as demographic information and RDT results were also collected on paper forms. These data were cross-checked twice with electronic data at the end of each day. R (version 3.4.0) was used for data cleaning and management and to produce summary statistics as well as graphics using the ggplot2 package. p values were calculated using Pearson *Χ*^2^ statistics with significance defined as *p *< 0.05.

## Results

The household survey included a total of 6485 participants. On average, 120 individuals were interviewed in each village. The outlet survey included 2 hospitals, 19 health centres, 39 dispensaries, 78 ADDOs, 57 drug stores, 9 pharmacies and 4 general stores or kiosks.

### Population characteristics in the household surveys

In the household survey, 4503/6485 (69.4%) participants were sampled in the two regions with moderately high malaria endemicity [2141/6485 (33.0%) in Mtwara and 2362/6485 (36.4%) in Mwanza] and 1982/6485 (30.6%) in the region with low endemicity (Mbeya) (Table 1). The majority of the participants lived in rural districts (4280/6485, 66.0%). The sample included 3623 (55.9%) females with a median age of 20 years (age range from 3 months to 95 years) and 2846 (43.9%) males with a median age of 14 years (age range from 3 months to 100 years). On the day of the survey, 1039/6485 (16.0%) participants reported a history of fever in the last 2 weeks prior the survey and 1136/6485 (17.5%) were tested positive for malaria by RDT.

**Table 1:** Population characteristics of participants in household surveys and febrile outpatients exiting health facilities

	Participants in household surveys (N = 6485)			Febrile outpatients, health facility exit interviews (N = 226)		
	N	%	95% CI	N	%	95% CI
Total	6485	100.0	–	226	100.0	–
Sex						
Male	2846	43.9	(42.9–45.0)	99	43.8	(38.4–49.2)
Female	3623	55.9	(55.0–57.0)	127	56.2	(50.8–61.6)
Missing	16	0.2	–	0	0.0	–
Age (years)						
0–4	1153	17.8	(17.0–18.6)	148	65.5	(60.3–70.7)
5–9	1014	15.6	(14.8–16.3)	48	21.2	(16.8–25.7)
10–14	775	11.9	(11.3–12.6)	9	4.0	(1.8–6.1)
15–24	990	15.3	(14.6–16.1)	6	2.7	(0.9–4.4)
25–44	1437	22.2	(21.4–23.1)	10	4.4	(2.2–6.7)
45–59	559	8.6	(8.1–9.2)	3	1.3	(0.1–2.6)
60–100	454	7.0	(6.5–7.65)	2	0.9	(− 0.1 to 1.9)
Missing	103	1.6	–	0	0.0	–
Area						
Urban	2205	34.0	(33.0–35.0)	50	22.1	(17.6–26.7)
Rural	4280	66.0	(65.0–67.0)	176	77.9	(73.3–82.4)
Missing	0	0.0		0	0.0	
Region						
Mwanza	2362	36.4	(35.4–37.4)	45	19.9	(15.5–24.3)
Mbeya	1982	30.6	(29.6–31.5)	37	16.4	(12.3–20.4)
Mtwara	2141	33.0	(32.1–34.0)	144	63.7	(58.5–69.0)
Missing	0	0.0	–	0	0.0	–
Had a fever in the previous 2 weeks^a^						
Yes	1039	16.0	(15.3–16.8)	100	–	–
No	5440	83.9	(83.1–84.6)	0	–	–
Don’t know	6	0.1	–	0	–	–
RDT result						
Positive	1136	17.5	(16.7–18.3)	106	46.9	(41.4–52.4)
Negative	5346	82.5	(81.7–83.3)	119	52.7	(47.2–58.2)
Missing	3	0.0	–	1	0.4	–
Took any anti-malarial drugs in the previous 4 weeks^a^						
Yes	810	12.5	(11.8–13.2)	NA^b^		
No	5664	87.3	(86.7–88.0)	NA		
Don’t know	11	0.2	–	NA		

^a^Based on self-report, ^b^ Not applicable

### Prevalence of *Plasmodium falciparum* and anti-malarials in the blood of the surveyed population

**Fig. 1:**
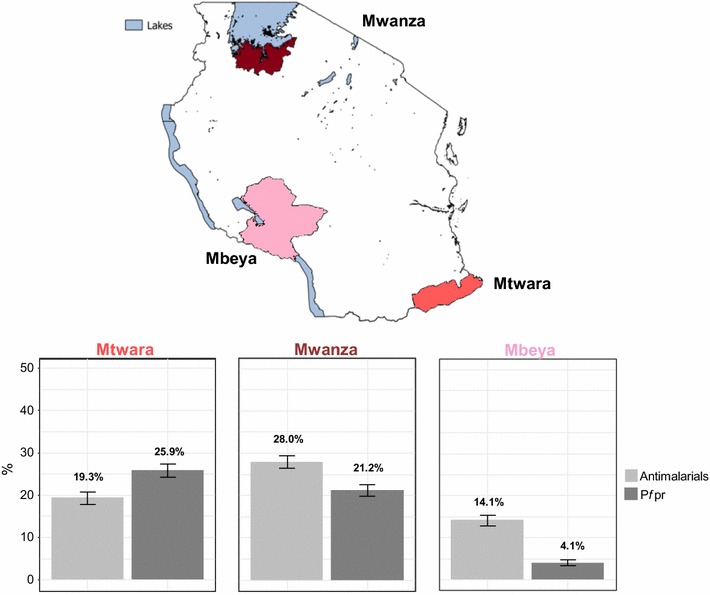
Proportions of individuals with residual anti-malarials in their blood and individuals with *Plasmodium falciparum.* The presence of anti-malarials in the blood was measured using dried blood spots samples and parasite prevalence using RDTs. These proportions were obtained from household surveys in three regions of Tanzania

Out of the 6485 participants, 94 were excluded from the anti-malarial analysis because their blood samples were not found or were mislabelled. Thus, 6391 participants remained with a valid anti-malarial measurement. Mtwara was the region with the highest malaria prevalence [25.9%, 95% confidence interval (CI) 24.4–27.5], followed by Mwanza (21.2%, 95% CI 19.8–22.6) and Mbeya (4.1%, 95% CI 3.4–4.9). The presence of anti-malarials was detected in the blood of 20.8% (95% CI 20.0–21.6, 1330/6391) of individuals in total. The proportion of individuals with residual levels of anti-malarials in the blood was 19.3% (95% CI 17.9–20.7) in Mtwara, 28.0% (95% CI 26.4–29.5) in Mwanza and 14.1% (95% CI 12.8–15.3) in Mbeya (Fig. 1).

### Reliability of medical history

**Fig. 2:**
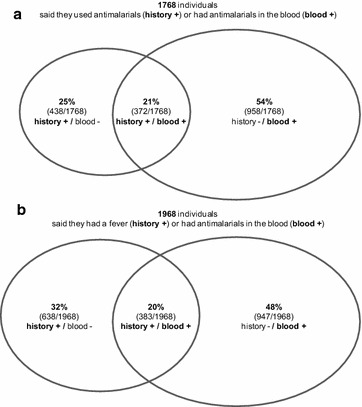
Overlap between self-reported history of anti-malarial use (**a**) or fever (**b**) and anti-malarials in the blood. Overlap between: (**a**) individuals reporting anti-malarial use in the previous month or (**b**) individuals reporting fever in the previous 2 weeks, and individuals with detectable concentrations of anti-malarial drugs in their blood (dried blood spots samples) in the household surveys

Out of the 1330 individuals with anti-malarials in their blood, only 28.0% (372/1330) reported the use of anti-malarials within the previous month, irrespective of fever status in the previous 2 weeks, as represented in Fig. 2a. There was only a 21.0% (372/1768) overlap between the individuals reporting anti-malarial use and those having detectable concentrations of anti-malarials in their blood.

Amongst the 6391 participants with a valid anti-malarial measurement, 16.0% (1021/6391) reported having had a fever in the previous 2 weeks. About 37.5% (383/1021) of them had detectable levels of anti-malarials in the blood (Fig. 2b). The overlap between the individuals reporting a fever and those having detectable concentrations of anti-malarials in their blood was 19.5% (383/1968). Participants who did not report any fever in the previous 2 weeks accounted for the majority of the individuals who had residual anti-malarial levels detected in their blood [71.2% (947/1330)].

### Seeking-care behaviour in case of fever

**Fig. 3:**
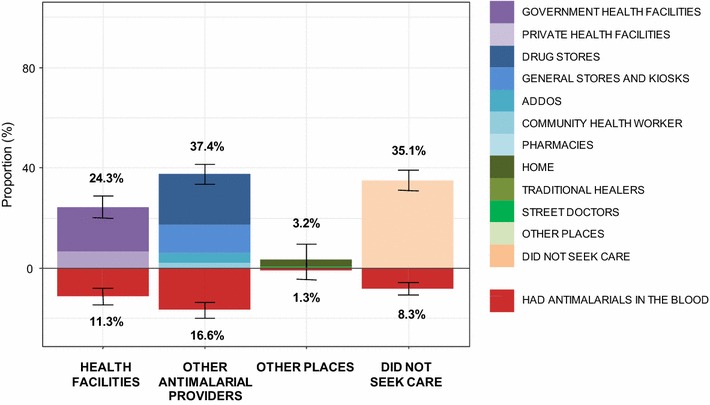
Prevalence of individuals with anti-malarials in their blood according to health-seeking behaviour. The top of the chart is based on the self-reported history of health-seeking behaviour of individuals who had a febrile episode in the previous 2 weeks. The bottom of the chart presents the corresponding prevalence of individuals with anti-malarial drug in their blood

Figure 3 presents the proportion of febrile individuals who sought care and those amongst them who had anti-malarials in their blood, according to the type of care providers they visited. Amongst the 37.5% (383/1021) of febrile individuals who had anti-malarials in the blood, the proportion who sought care in health facilities was lower than in non-health facility anti-malarial providers [11.3% (115/1021) vs. 16.6% (170/1021)]. 8.3% (84/1021) said they did not seek care and 1.3% (13/1021) reported seeking care by a friend or by a traditional healer or took a drug from their home.

### Appropriateness of malaria diagnosis (according to medical history) and treatment (based on anti-malarials in the blood) in health facilities and non-health facility anti-malarial providers

**Fig. 4:**
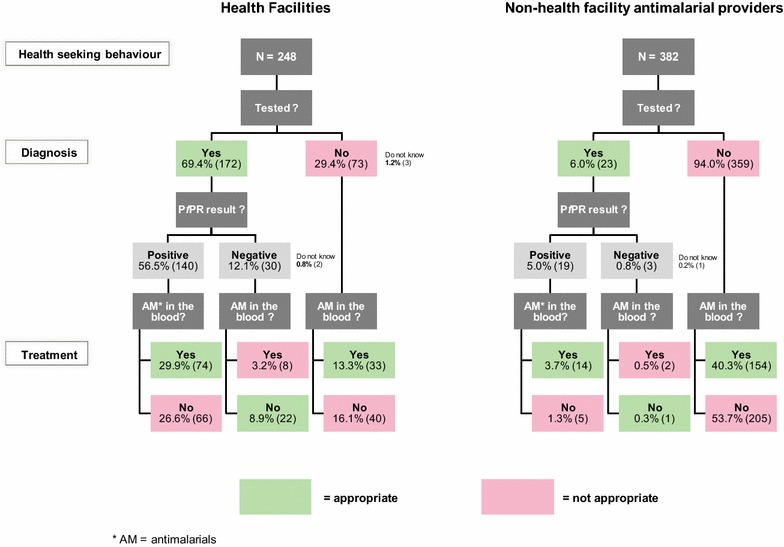
Proportion of febrile individuals appropriately diagnosed and treated for malaria. Appropriate diagnosis was defined as a patient with history of fever being tested for malaria (by RDT or microscopy) and appropriate treatment as having anti-malarials in the blood if the RDT result was positive or if the person had not been tested. The left side of the figure reports individuals who sought care in health facilities and the right side of the figure those who sought care in non-health facility anti-malarial providers. The upper part of the organigram is built upon self-reported history and the bottom part on results of anti-malarials measured in blood samples collected during the household survey, which constitute more objective information

As shown in Fig. 4, proportion of participants with fever who reported they had been tested for malaria at the place they sought care was 31.0% (195/630), with a large statistically significant difference between health facilities and non-health facility anti-malarial providers [69.4% (172/248) vs. 6.0% (23/382), respectively; *p *< 0.001)]. The overall proportion of people being appropriately treated was 47.3% (298/630). This proportion was 52.0% (129/248) in health facilities vs 44.2% (169/382) for non-health facility anti-malarial providers (*p *= 0.04). Only half of the individuals who were tested positive by RDT in the health facilities had anti-malarials detected in their blood [52.9% (74/140)]. This was close to those who were not tested [45.2% (33/73) in health facilities and 42.9% (154/359) in non-health facility anti-malarial providers]. One-third of the individuals tested negative in the health facilities had detectable levels of anti-malarials in their blood [26.7% (8/30)]. Overall, the proportion of individuals treated was larger than that being tested [45.2% (285/630) treated, 31.0% (195/630) tested].

### Appropriateness of malaria treatments assessed by medical history and anti-malarials in the blood in household surveys

**Fig. 5:**
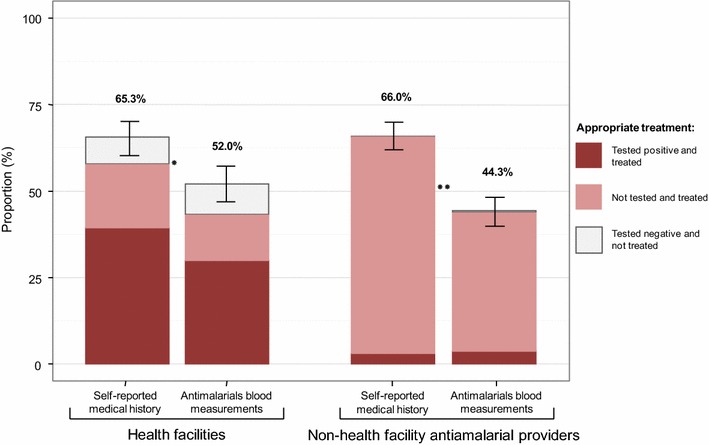
Comparison between appropriateness of treatment assessed according to self-reported medical history and anti-malarials blood measurements. The proportions of febrile individuals interviewed in household surveys appropriately treated for malaria when they sought care in health facilities (left side of the figure) and non-health facility anti-malarial providers (right side of the figure) are assessed according to self-reported medical history and anti-malarials blood measurements. There is a significant difference (*p < 0.01, **p < 0.001) between the proportion of febrile patients appropriately treated for malaria assessed according to self-reported medical history and anti-malarials blood measurements

When assessed based on participant’s medical history recorded in household surveys, the proportion of individuals who mentioned being appropriately treated according to diagnostic test result or who stated being treated presumptively was significantly higher than when it was assessed on the basis of presence or absence of anti-malarials in the blood, as shown in Fig. 5 (65.3% vs. 52.0%, *p *< 0.01 in health facilities and 66.0% vs. 44.3%, *p *< 0.001 for non-health facility anti-malarial providers).

### Availability of anti-malarial treatments and RDTs in each region

Mbeya was the only region in which all health care providers (health facilities and non-health facility anti-malarial providers) had anti-malarials in stock (Table 2). The availability of malaria blood testing (by RDT or microscopy) was the highest in Mbeya in all types of care providers (88.9% in health facilities and 7.9% in non-health facility anti-malarial providers). 68.8% of the health facilities in Mtwara and 80.8% in Mwanza had malaria diagnosis tools available. Very few non-health facility anti-malarial providers had RDTs in stock in these two regions (0.0% in Mtwara and 1.1% in Mwanza). In both types of care providers and in each region, the commodities to treat were higher than the potential to test for malaria.

**Table 2:** Proportion of screened outlets with anti-malarials and malaria diagnosis tools available in stock

	Mbeya				Mtwara				Mwanza			
	Health facilities (N = 18)		Non-HF AM providers (N = 38)^a^		Health facilities (N = 16)		Non-HF AM providers (N = 21)		Health facilities (N = 26)		Non-HF AM providers (N = 89)	
	N	% (95% CI)	N	% (95% CI)	N	% (95% CI)	N	% (95% CI)	N	% (95% CI)	N	% (95% CI)
% of outlets with anti-malarials in stock	18	100.0	38	100.0	16	100.0	20	95.2 (87.6–102.9)	26	100.0	81	91.0 (86.0–96.0)
% of outlets with RDTs in stock or microscopy	16	88.9 (76.7–101.1)	3	7.9 (0.7–15.1)	11	68.8 (49.7–87.8)	0	0.0	21	80.8 (68.1–935.)	1	1.2 (− 0.71–2.96)

^a^Non-HF AM = non-health facility anti-malarial. This table reports stocks available on the day of the survey. Health facilities include hospitals, public and private health facilities and dispensaries. Non-health facility anti-malarial providers include pharmacies, drug stores, ADDOs and general stores. Malaria diagnosis tools include RDTs and microscopy

### Exit interviews

A total of 456 outpatients were interviewed in 37 nearby health facilities (3 hospitals, 17 health centres and 17 dispensaries), but for the present analysis, only the 226 febrile patients were considered (Table 1). The proportion of interviewees that reported they had been tested at the health facility was 65.9% (149/226), a proportion that is close to that obtained through household surveys [69.4% (172/248)]. 65.0% (147/226) had received appropriate treatment, a proportion that is higher than that in household surveys, although this difference was not statistically significant (*p *= 0.16). During the interviews, 46.9% (106/226) febrile participants were tested positive by RDT by the field investigators, a proportion which is lower than that reported through household surveys [81.4% (140/172), *p *< 0.01].

## Discussion

According to relevant literature, this is the first study investigating the presence of anti-malarials in the blood of the general population. The measurement of nine anti-malarials provided a reliable endpoint and allowed a comprehensive assessment of drug use and current malaria case management landscape in the studied communities.

This study showed that close to one-fifth (20.8%) of individuals in the community had residual anti-malarials in their blood, even in Mbeya, a region of low endemicity of malaria. The absence of relationship between the level of transmission and the drug-prescribing behaviour has also been observed in another study in Tanzania [28]. The high prevalence of individuals with anti-malarials in the blood in Mwanza and Mbeya in comparison to the prevalence of *Plasmodium falciparum* does not seem to be related to low testing available since these two regions had higher proportions of health care facilities with malaria diagnostic tools in stock. However, having diagnostic tools available does not guarantee their usage, or that clinicians are compliant to tests results. The testing habits might be lower in these two regions, and especially so in Mbeya where the probability of a fever being malaria is much lower than in Mwanza or Mtwara because of the higher altitude [22].

More access to drugs might be one explanation. Indeed the study team was able to visit 115 nearby outlets in Mwanza, 56 in Mbeya, but only 37 in Mtwara for roughly the same number of individuals in the vicinity. Mwanza was the region with the highest proportion of individuals with anti-malarials in the blood, and also the highest prevalence of fever (34.0% in Mwanza vs. 14.6% in Mtwara and 9.3% in Mbeya). More febrile episodes in Mwanza could be due to a higher prevalence of other causes of fever (e.g. arboviruses) which might be wrongly allocated to malaria [29]. Alternatively, the population of Mwanza might have a lower level of malaria immunity than that of Mtwara, with a proportion of *Plasmodium* infections that progress to clinical manifestations being higher in this setting of lower endemicity, and hence higher prevalence of individuals treated with anti-malarials [30]. The study results confirm this hypothesis with 35% of the individuals tested positive for malaria in Mwanza reporting having had a fever in the previous 2 weeks, against only 23% in Mtwara.

Whatever the reasons are for the high prevalence of people with anti-malarials in the blood, access to drugs does not seem to be a major issue in these communities, but rather appropriate case management to target those febrile patients that are sick with malaria. The important drug pressure across these three regions is worrying because the occurrence of a low drug level in blood induces strong selective pressure on parasites and causes the emergence of drug resistance [11, 31].

When using an objective endpoint such as the concentration of anti-malarials in the blood, it appears that self-reporting of drug intake is unreliable, with only 28% concordance for individuals having detectable levels of anti-malarials in their blood reporting the use of such treatments within the previous month, and 54% pretending having taken any drug actually having no residual anti-malarials in their blood. Poor agreement between history taking and anti-malarial concentration measurement has already been shown in two previous small-scale studies that were conducted among Tanzanian and Cambodian patients attending a health facility [17, 18], and in a cross-sectional survey in Uganda [32].

Amongst the individuals who had detectable levels of anti-malarials in their blood, 71% said they did not have fever in the previous 2 weeks and 6% said they had fever but did not seek care. Again, these results show that history taking is very unreliable and that all previous studies that described health seeking behaviour and drug consumption [12, 13, 33] should be taken with caution, as they leave aside a considerable part of the population using drugs. Although it has been shown that history validity can be improved [34], new technology platforms such as LC–MS/MS performed on DBS samples allow to move from subjective to objective and reproducible data. There is obviously a question of feasibility (ideally the DBS should be stored frozen) and cost (50 dollars for measurement of nine different anti-malarials by LC–MS/MS in one DBS sample) to conduct large-scale surveys, but certainly such objective assessment could be used as a validation method for other more practical tools.

Another main finding of this study is the poor diagnosis and treatment practices in case of febrile episode. Overall, only one-third of the febrile individuals being tested for malaria at the place they sought care and about half being appropriately treated according to their diagnostic test result, or presumptively if no diagnostic tool was available. Presumptive treatment is thus still common, and far from the goal set by the WHO of systematic testing of suspected malaria cases and treatment upon result [3]. The low testing rate is mainly due to the high proportion of individuals seeking care outside the health facilities where patients are usually not tested. Allowing drug retailers to perform malaria testing might be one way of reducing the numbers of anti-malarials sells and consumption. In Tanzania, an ongoing research in which dispensers from ADDOs in intervention districts are trained to perform RDTs and treating with first-line treatment has already shown encouraging results with an increase from 0 to 65% of suspected malaria patients visiting a shop being tested [10].

The proportion of febrile individuals appropriately tested for malaria was much higher in health facilities than in non-health facility anti-malarial providers (70% vs. 6.0%), but this difference was not much reflected in a better targeting of treatment to individuals with malaria. Indeed, the proportion of patients appropriately treated was only eight percentage point higher in health facilities than in non-health facility anti-malarial providers [52.0% (129/248) vs. 44.2% (169/382), *p *= 0.04]. This can be partly explained by the fact that, according to anti-malarial drugs detected in the blood of the interviewees, only half of the patients tested positive were treated. The same proportion of anti-malarial drugs was detected in the blood of patients who were not tested.

In the literature, a decrease by up to three-quarters in ACT prescription has been observed after RDTs implementation, between 2006 and 2008 [35, 36]. A recent systematic review including 14 studies showed that the overall compliance to positive and negative RDT results was 97 and 78%, respectively, and that lower levels of health care workers complied better that the more professional counterparts [37]. The results of the present study are less encouraging since the fever cases were not always tested and appropriately treated. Studies rolled out in places where training has been done are more likely to detect a positive effect and a change observed at a given time point might not be sustainable. Besides, the study findings add to previous household surveys conducted in the same regions 3 years before which showed no significant change in the proportion of febrile individuals obtaining an anti-malarial at the population level [5, 6]. This was explained by a reduction in the use of health facilities. Indeed, ADDOs are now recognized to be the principal source of medicines in Tanzania [38].

These results call for interventions to reinforce the whole system for a public health impact. Health facilities priorities should focus on improving systematic testing and treating all positive cases. As for non-health facility anti-malarial providers, our findings support the suggestions already drawn from two recent trials that introducing RDTs into regulated private sector settings can improve malaria testing and reduce over-treatment [10, 39]. Testing and treating should also be encouraged at low level of health care, e.g. at the level of community health workers, as it has been proven to improve well-targeted ACT in randomized cross-over trials in Africa [40, 41].

In addition, care should be taken when assessing appropriateness of treatment in the population. This study observed a higher proportion of appropriate treatment when assessed by self-reported medical history than by anti-malarials measured in the blood and there are several potential reasons to explain such discrepancies: in case of a positive test result, the treatment received may have not been an anti-malarial or patient might have poorly adhered to their treatments [19, 42, 43]. In the case of a negative result, patients could have obtained a treatment from the informal sector and not reporting it.

Exit interviews confirmed the findings of the household surveys. Only 58% of the patients tested positive by the field investigators prior leaving the health facilities had received treatment against malaria during their consultation. The most likely scenario is poor compliance by health professionals to an RDT positive result and no presumptive treatment prescribed to fever cases not tested. In contrast, a rather small proportion of the patients tested negative received a treatment (11%), which indicates a good compliance to negative results. Such findings imply that if more febrile patients would be tested, the amount of unnecessary treatment distributed would decrease.

The present study has several limitations. The history of fever recall was based on a 2-week period in order to be able to compare with previous surveys in the same areas, but residual anti-malarial drugs can eventually be detected in the blood for up to 4 weeks. This could explain why a proportion (71%) of individuals who did not report a fever in the previous 2 weeks had detectable levels of anti-malarials in the blood. However, as already mentioned before, history of drug intake was made on a 4-week period record in order to make the comparison between self-reported use and blood levels of anti-malarials possible. When assessing reliability of medical history, the direct comparison of history of drug intake based on a 1-month recall-period and drug measurements is limited by Lume fantrine important inter-individual variability [44] and by individuals’ treatment adherence rate [43]. The evaluation of diagnosis appropriateness from household surveys was based on self-reported medical history only, and thus subjected to report bias. This limitation is nevertheless inherent to all studies of this kind, except for those that rely on direct observation of the consultation, which are subject to the Hawthorne effect though. Finally, this study did measure diagnosis and treatment practices in different settings (urban or rural) and different transmission areas, but only at one point in time.

## Conclusion

Despite recent efforts that have been made to improve access to diagnostic tools and to reduce anti-malarials overuse, there is still a considerable anti-malarial drugs pressure at the population level. Improving rational use of drugs is necessary to prevent the development of resistance. The present findings indicate that the goal of the WHO guidelines of systematic diagnostic testing and treatment upon result is far from being reached, and that anti-malarial treatments are not targeted to the individuals in need. As resources are invested into the development and implementation of new diagnostic tools and effective treatments, it is of paramount importance to make sure that those tools are used to their full potential, and properly enforced. New health care interventions should not only be evaluated for their impact at the level of health facilities, but also at the level of the community. However, household surveys that collect information on health-seeking behaviour or practices through self-history are subject to important biases. Evaluation or monitoring tools that rely on objective measurements such as drug concentration in the blood should be favoured, if feasible.
